# Germline genetic profiling in prostate cancer: latest developments and potential clinical applications

**DOI:** 10.4155/fso.15.87

**Published:** 2015-12-18

**Authors:** Mahbubl Ahmed, Rosalind Eeles

**Affiliations:** 1 The Institute of Cancer Research, London SM2 5NG, UK

**Keywords:** clinical application, genetic variation, genome-wide association study, prostate cancer, risk modeling, single nucleotide polymorphism

## Abstract

Familial and twin studies have demonstrated a significant inherited component to prostate cancer predisposition. Genome wide association studies have shown that there are 100 single nucleotide polymorphisms which have been associated with the development of prostate cancer. This review aims to discuss the scientific methods used to identify these susceptibility loci. It will also examine the current clinical utility of these loci, which include the development of risk models as well as predicting treatment efficacy and toxicity. In order to refine the clinical utility of the susceptibility loci, international consortia have been developed to combine statistical power as well as skills and knowledge to further develop models that could be used to predict risk and treatment outcomes.

## Abstract (lay abstract)

Prostate cancer is one of the most common cancers to occur in men in the western world. The incidence of prostate cancer is however lower in Asian countries. There are some families who have many male members affected with prostate cancer. The incidence of prostate cancer is also increased in men of Afro-Caribbean ancestry. Studies have shown that there are inherited genetic changes that may be accounting for these differences, however only one third of these changes have been discovered so far. This review aims to discuss how these genetic changes have been discovered and how they can potentially be used in every day clinical practice.

**Figure 1.**
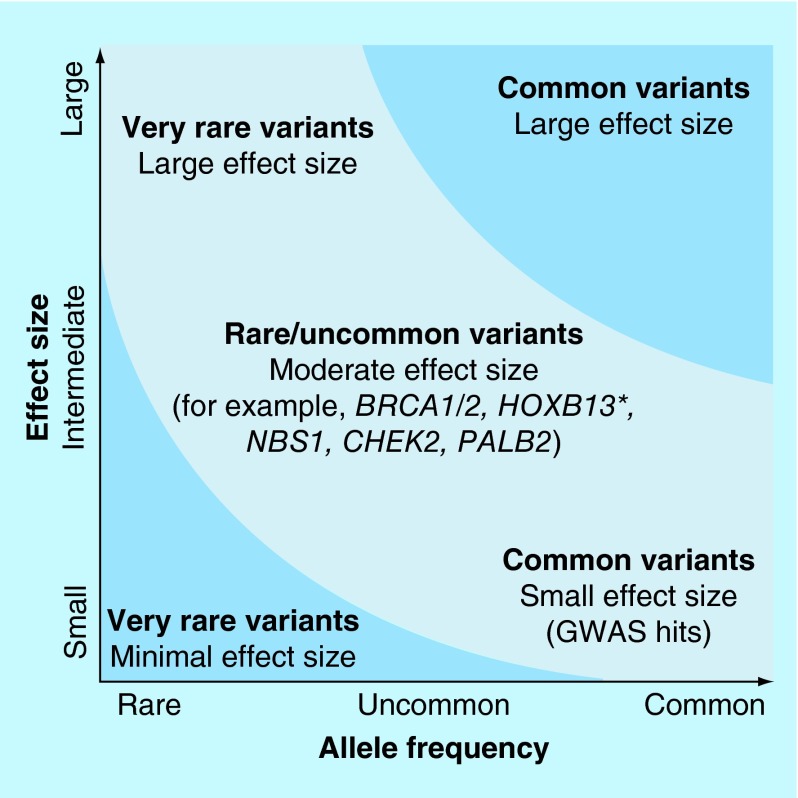
Diagram above showing that prostate cancer susceptibility is likely to be due to a mixed model of common and rare variants [83]. Permission obtained from Nature Publishing Groups.

**Figure 2.**
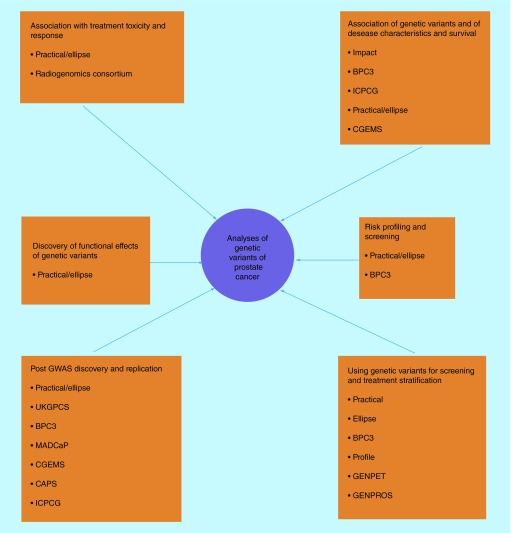
**Showing members of the consortia who are involved with investigating the role of genetic variants of prostate cancer.** It highlights the collaborative efforts needed to answer the various scientific and clinical questions. BPC3:Breast Prostate Cancer Cohort Consortium [139]; CAPS: Cancer in the Prostate in Sweden [140]; CGEMS: The Cancer Marker Susceptibility Projects [141]; ELLIPSE: Elucidating Loci Involved in Prostate Cancer [142]; GWAS: Genome Wide Association Studies; GENPET: An imaging study of FCH-PET-CT in men with prostate cancer and a BRCA gene mutation (Study in preparation) (Research Ethics Number: 15/20/0242); GENPROS: Analysing outcomes after prostate cancer diagnosis and treatment in carriers of rare germline mutation in cancer predisposition genes. (Study in preparation) (Research Ethics Number: 14/20/0072); ICPCG: International Consortium of Prostate Cancer Genetics [143]; IMPACT: The Identification of Men with a Genetic Predisposition to Prostate Cancer: Targeted Screening in BRCA1/BRCA2 mutation carriers and controls [144]; MADCaP: Men of African Decent and Prostate Cancer [145]; PRACTICAL: Prostate Cancer Association Group to Investigate Cancer Associated Alterations in the Genome [146]; PROFILE: Germline genetic profiling: Correlation with targeted prostate cancer screening and treatment [147].

Prostate cancer has become the second most prevalent cancer affecting men worldwide, and the fifth most common cancer overall [1,2]. In 2008 there were 899,000 new cases of prostate cancer diagnosed worldwide with more than two-thirds being diagnosed in the western world [1]. Globally there is huge variability of incidence, with western countries having a higher incidence compared with the Far East and the developing world [3]. There is up to 25-times variation in prostate cancer incidence as compared with variation in mortality which is up to tenfold. This is most likely due to the use of prostate specific antigen (PSA) testing, which impacts more on incidence than mortality [1,2]. Epidemiological studies of Koreans migrating from Korea to the USA have shown that the incidence of prostate cancer among the Korean immigrants is similar to that of the local American population rather than native Koreans [4]. These results suggest that environmental factors may have an impact in the incidence of prostate cancer. However these risk factors have so far not been identified in the development of prostate cancer.

The only established risk factors for prostate cancer are that of advancing age, race and family history [5]. Age-specific incidence rates of prostate cancer in the UK rise steeply from age 50 to 54 reaching a maximum at age 75–79, and 75% of all cases diagnosed are in those aged over 65 [2]. African–Americans have the highest incidence of prostate cancer (275.3 per 100,000 men) which is 60% higher than an age matched Caucasian population (172.9 per 100,000 men) [5]. This high incidence has also translated into an increased death rate of African-Americans from prostate cancer [5]. Family history remains the most well studied/investigated prostate cancer risk factor. For men who have a first degree relative (father/brother) affected with prostate cancer, their risk of developing prostate cancer is double that of the general population risk [6]. Those with a positive family history usually present at an earlier age, and have a higher relative risk if more than one first degree relative is affected [7]. This relative risk is four-times that of the general population, if the first degree relative affected is below 60 years of age [7]. Twin studies using data from cancer registries in the Nordic region have shown a 50% higher risk of prostate cancer in monozygotic twins compared with dizygotic twins [8,9]. This study suggests that it is likely that shared genetic factors may contribute to the risk of prostate cancer rather than shared environmental factors. It is thought that germline mutations may account for 42–58% of prostate cancer risk [9].

There is epidemiological evidence to suggest that diet may have an influence on prostate cancer incidence. A report by the World Cancer Research Fund/American Institute for Cancer Research reported that diets rich in foods containing lycopene (found in tomatoes) or selenium have a protective effect in the development of prostate cancer [10]. Follow-up studies investigating the role of lycopene have shown increased lycopene intake with a reduced incidence of prostate cancer development [11]. The SELECT trial, which was a randomized controlled trial, showed that selenium supplementation did not reduce the incidence of prostate cancer development [12]. However the difference in the action of Selenium may be due to its availability directly from the diet rather than through supplementation [13]. Reports have also suggested other dietary factors that may have a protective element in the development of prostate cancer. These include cruciferous vegetables, vitamin E, polyphenols, soy/isoflavones, omega-3 and coffee [14–16]. Dietary factors that are thought to increase the risk of prostate cancer development are increased circulating folate levels, increase red meat and saturated fat intake [14–15,17]. Environmental factors that interact with diet may also have an influence on prostate cancer incidence. Reports have suggested that increased early life sun exposure and hence vitamin E levels may also be a protective factor for prostate cancer development [18].

This review aims to review the current evidence linking germline genetic variants with various clinical parameters of prostate cancer, including stratification of risk of prostate cancer and response to treatment and treatment toxicity.

## Early genetic links

The early studies focused on the fact that familial clustering of prostate cancer may be linked by simple Mendelian inheritance patterns. Mendelian patterns of inheritance are more likely to be due to genes which are highly penetrant but which are rarely found in the genome. Intermediate and low penetrant genes are more likely to be commonly found in the genome. Segregation analysis uses familial data to predict the mode of inheritance of a genetic variant. Segregation analysis of families with prostate cancer suggested that a gene which is highly penetrant may be the underlying cause of clustering within families. Inheritance models such as autosomal dominant, recessive and X-linked were suggested as some of the modes of transmission of genetic predisposition to prostate cancer [19–24]. The major drawback of segregation analysis in predicting the mode of inheritance was that the disease is common and so cases can occur which are not due to the variant (so called phenocopies) which lowers the statistical power to detect a true association.

Linkage analysis is a gene mapping method that aims to identify genetic variants in familial clusters. It uses co-segregation as the method of analysis which is the co-inheritance of a genetic marker with a known phenotype, in this case, prostate cancer. Several genes have been suggested as candidates for hereditary prostate cancer such as *ELAC2, RNASEL* and *MSR1* [25–27]. Unfortunately none of these genes that have been suggested as possible candidates have been replicated by other groups.

The International Consortium of Prostate Cancer Genetics (ICPCG) is a group that was established to identify prostate cancer susceptibility genes through the combined analysis of linkage data from familial clustering of prostate cancer. In 2005 they published a genome-wide screen for prostate cancer susceptibility genes in 1233 prostate cancer families from ten international groups [24]. In the combined analysis five regions were identified which were suggestive of linkage with a LOD (logarithm to base of 10, of odds of linkage) score >1.86. A subsequent subset analysis was performed from families which had more than five family members affected, and who were also young at presentation [24]. The results of this study showed that there was potentially a prostate cancer susceptibility gene on chromosome 22q12, which had a LOD score of 3.57. Several other regions had lower LOD scores, suggestive of linkage.

## Association studies

In the search for susceptibility genes one of the biggest limitations was designing studies that were large enough to detect genetic variants with small effects that are common and thereby important for population risk profiling which are associated with prostate cancer. This was overcome by designing large association studies. Association studies compare the frequency of genetic variants in patients with the disease (cases) versus patients without the disease (controls). It is analogous to a large game of spot the difference using the genome of the two groups. Once a genetic variant has been discovered, statistical tests are undertaken to confirm that the difference between the frequencies in the two groups is not a chance finding. Once a specific genetic variant (allele) is identified, it is then quantified or classified by its effect size, in other words, what increased risk of prostate cancer does an individual have if they possess this variant compared with members of the population who do not. The effect size is measured by the ratio of the frequency of the allele in cases to the ratio of the allele in the controls. This is also known as the odds ratio. If the allele is more common in cases than controls than the odds ratio would be greater than 1. A Chi-squared test is commonly used to calculate the probability of rejecting the null hypothesis. The overall aim of genetic association studies is to find genetic variants that confer an increased risk of prostate cancer and therefore have an odds ratio greater than 1. The initial focus of researchers was to focus on alleles with an odds ratio greater than 2 and therefore a large effect size. These genes with a large effects size are more likely to cause the phenotype and therefore be more penetrant but occurred rarely in the population. It soon became apparent than common alleles with smaller effect sizes were more prevalent in the population and in combination could increase the risk of developing the phenotype of prostate cancer [28].

The most common genetic variants in the human genome are single nucleotide polymorphism (SNP) [29,30]. SNPs are variations in DNA sequences when a single nucleotide differs between members of related DNA strands. An example is as follows: AGGCGAT to AGGCGCT; the base adenine found more commonly in the population is replaced with cytosine which is known as a SNP and occurs as a minor allele (less frequent) in the population. SNPs occur randomly throughout the genome and generally are found more frequently in non-coding regions than coding regions of DNA. Initially investigators focussed on the SNPs that were found within coding regions, as the thought was that an altered protein could have downstream effects, such as activation of an oncogene or deletion of a tumor suppressor gene and therefore lead to a disease trait such as prostate cancer. This type of association study is known as a candidate gene association study [31]. Candidate genes are identified by using known genes involved in prostate cancer. These genes are molecularly profiled in cases and controls to identify genetic variants associated with prostate cancer. This approach has yielded several interesting candidate genes such *NBS1, CHEK2* and *PLAB2*, but these have yet to be validated in other studies and other ethnic groups [32–37]. The candidate gene approach was initially very popular based on the cheap cost to perform them, but unfortunately these studies have resulted in very few positive results and have been very disappointing. One of the main issues with the candidate gene approach is the small sample size in the studies, which made it difficult to detect variants with smaller effect sizes. The other major drawback with the candidate gene approach is that it is restricted to genes whose function is established and therefore selective in terms of what part of the genome is assessed. In order to look for these variants with small effect sizes a more nonselective method was required for gene discovery.

In the late 1990s, researchers agreed that for complex diseases such as cancer, multiple genes were likely contributing (polygenic) to the cancer phenotype [38,39]. A new method of genetic variant discovery was needed, which could involve a large scale association study which could be more efficient and effective in the discovery phase. This type of association study is known as the genome wide association study (GWAS).

## Genome wide association studies (GWAS)

As like other association studies, GWAS consists of large number of cases and controls in order to achieve the power required to detect statistical significant effect sizes. Unlike the candidate gene approach, GWAS allows discovery of new variants in a nonselective (agnostic) way with the ability to assay 300,000 to 5 million SNPs concurrently [31]. The statistical analysis is performed by comparing alleles that are present more commonly in cases than controls. If an allele is present more frequently in cases than controls then an estimated effect size can be calculated. This effect size is then presented as an odds ratio, in other words, the odds of developing prostate cancer if the SNP is present in cases compared to the odds of not having the disease if that SNP is a controls. Initially at first investigators focused on allele odds ratio of greater than 2. Statistical significance or p value is calculated by dividing the significance level usually 0.05 by the number of statistical tests performed. For example, if 1 million SNPs are investigated in a GWAS, 1 million statistical tests are undertaken thus resulting in the significance level being 1 × 10^-8^, which is also known as genome wide significance. Currently p < 5 × 10^-8^ is considered genome-wide significant.

## SNPs & variation in the genome

Whole genome sequencing technology has been able to quantify the magnitude of genetic variation between individuals, which is estimated to be less than 0.5% which equates to several million differences between individuals. Of these differences, single base substitutions which result in SNPs are the most common. Other DNA structural abnormalities that lead to larger structural alterations or change in the number of genes expressed (due to addition or deletion of genes known as copy number variants) has been shown by next generation sequencing analysis to affect more bases across the genome but to occur less frequently [40]. The mean allele frequency (MAF) describes the frequency at which a SNP is present in a reference population. The MAF of SNPs can vary depending on the reference population that is assessed. For example SNPs common in a European population may not be common in an African population. Common SNPs are defined as those with a MAF >5%, and so far 4 million have been identified in the 1000 Genomes project [41]. Rare SNPs are defined as having a MAF 1–5% and it is estimated that there are 10 million of these [42]. Of the total number of SNPs present in the population approximately 250,000 may be present in coding regions of DNA which are thought to alter protein production and function [43]. These SNPs in coding areas are called cSNPs and are divided into nonsynonymous SNPs which do alter protein function and synonymous SNPs which do not. Much effort has been spent trying to associate these nonsynonymous SNPs with the biological cause of disease but very little association has been found [43]. The majority of the cSNPs is in noncoding regions and their full function is not known [44]. It has been postulated that these SNPs may control gene expression by having an effect on gene promotor or enhancer elements [44]. Despite the greater understanding of the potential functional effects of the SNPs further work needs to be undertaken in this area [45].

It is now technically possible to individually genotype every known SNP to test the association with any disease trait. However due to the large numbers of SNPs available this may not be practical or economically efficient. Genotyping studies have shown that many SNPs are not just inherited individually but also with segments of chromosomes with neighboring SNPs when passed down from generations [30,46]. Therefore SNPs can be inherited with other SNPs, known as tag SNPs, and if this is the case then these SNPs are thought to be in linkage disequilibrium (LD). Tag SNPs can sometimes be in LD with more than one SNP. This link means that one tag SNP can give information about many adjacent SNPs and therefore make genotyping more efficient and cost effective. To cover the European population, approximately 500,000 SNPs are required for complete GWAS coverage [47]. More SNPs would be required to cover those with an African history due to previous mixture of Europeans with Africans [48].

## Variants discovered from GWAS studies

There have been 25 published prostate cancer GWAS since 2006, in which the genetic findings have been published at the NIH website [49]. The combination of all the GWAS has identified 100 published Prostate cancer SNPs, which are shown in Supplementary Table 1 [47,50–72]. One the earliest regions discovered was 8q24 which has documented independent genetic variants associated with prostate cancer, the highest number so far discovered [66,72–74]. 8q24 has potential important clinical implications as it located near the *myc* proto-oncogene and may act as an enhancer of this region by interacting with chromatin conformation [73].

Another important SNP, rs10993994, is located on chromosome 10 which is located 2bp upstream of the transcription initiation site of microseminoprotein-β (*MSMB*) [75]. *MSMB* codes for a protein secreted in the prostate gland called PSP94, which is exclusively produced in the prostate and secreted in the semen. PSP94 is a protein that is thought to be involved in the regulation of growth and also involved in apoptosis of prostate cancer cells, and can be measured in the plasma when it is released from prostate cancer cells [76]. It has also been shown that reduced levels of PSP94 are correlated with progression after radical prostatectomy, and also experiments have shown that the protein is lost early in prostate cancer development [77,78]. Therefore potentially PSP94/MSMB could be used as a biomarker in combination with PSA, this will however need to be validated [79].

SNPs have also been associated with PSA expression in the region of chromosome 19. In this region there are genes which encode for a sub group of serine proteases called Kallikreins to which PSA belongs. SNPs close to the genes of kallkirein-related peptidase 2 (*KLK2*) and *KL3* (PSA) have been shown to increase the risk of prostate cancer and increase the levels of PSA [80]. Fine mapping has been undertaken in the region around 19q13 which has led to the discovery of a SNP called rs2735839 near the *KLK3* gene which affects PSA production [81]. If this SNP is present then the serum PSA measured at diagnosis may not be accurate in staging the patient's prostate cancer. Some of the Kallikreins are used in prognostic models with other factors such as free and total PSA to predict risk of cancer [82]. More work needs to be done before this can be applied to prostate cancer screening. There may be potential therapeutic options using SNPs. One of them is a SNP rs51919432 which is near the androgen receptor gene and could be a target for therapy [66]. SNPs have also been discovered in prostate cancer which have also been associated with other diseases. A SNP called rs130067 is found in prostate cancer as well as psoriasis, which may mean that there may be similar pathways in the two diseases which could potentially have diagnostic and therapeutic benefits [24,66].

It is thought that the genetic architecture of prostate cancer is due to a mixed model of common and rare variants. This is demonstrated in Figure 1 [83].

## Rare variants

These are variants which have a minor allele frequency of <5%, and occur too infrequently to be detected on a GWAS. They are also inadequately tagged by other SNPs to be genotyped by common arrays used. Very large numbers of cases and controls are needed to detect these rare variants. Next generation sequencing of targeted areas or whole genome sequencing has enabled the detection of these rare variants. In the late 1990s the Breast Cancer Linkage Consortium (BCLC) published results in which they analyzed men in families in which the females developed breast and ovarian cancer caused by *BRCA* mutations. The results showed that men have a relative risk of prostate cancer of five in those who have a germline *BRCA2* mutation compared with men without a mutation. This relative risk increases to up to seven-times if the men in the family develop prostate cancer below the age of 65 [84]. A further larger study was undertaken which screened 2000 men with prostate cancer. This showed that just over 1% of men who developed prostate cancer below age of 65 carried a deleterious *BRCA2* mutation and often they did not have a positive family history [85]. For men who are carriers of a *BRCA1* mutation, data from the BCLC, and other studies have shown that there is an approximately four-times relative risk of developing prostate cancer for the under 65 compared with those without the mutation [86]. In a recent somatic sequencing study of castration resistant prostate cancer the germline *BRCA2* mutation rate was shown to be as high as 12.7% [87].

Several groups have shown that *BRCA1* and *2* mutation carriers have a more aggressive form of prostate cancer than those without the mutation, and also have a worse prognosis [88,89]. Mutation carriers are also likely to present with higher risk of local nodal involvement as well as distant metastatic disease, which again translates to a poorer survival outcome [90]. The optimal radical treatment option for these patients is yet to be determined, but radical prostatectomy is thought to be the most suitable treatment option [91]. This potentially could be a very important therapeutic tool as those who develop prostate cancer with a *BRCA* mutation could undergo more personalized intensive treatment.

Another rare variant has been discovered, which is present in a gene called *HOXB13*, and belongs to a class of genes which encode transcription factors in the homeobox family. The germline genetic mutation is found in the coding region of the *HOXB13* gene, and is known as G84E. Mutations in the *HOXB13* gene have been shown to be associated with prostate cancer development in mouse models and more specifically an androgen-independent phenotype [92,93]. In a large study performed by the ICPCG consortium, 5% of Europeans with prostate cancer carried the *HOXB13* variant, and it is particularly prevalent in Nordic countries where the frequency can be as high as 22% [94]. At present it is uncertain if prostate cancer due to germline mutations in *HOXB13* is more or less aggressive than prostate cancer not due to such mutations and such clinical data will determine how such patients should be screened and managed.

## Creating genetic profiles & risk models

In order to utilize the discovery of germline genetic variants, the results of the gene finding experiments need to be translated into useful clinical applications. Risk profiling and models are being developed where the current known germline genetic mutations are used to predict the risk of developing prostate cancer. The main limitation of this is that it is predicted that only 33% of common germline genetic variations are known at present [95]. Therefore it may not be very reliable to create a risk model when the majority of variants of prostate cancer are not known, however this could potentially change with the discovery of new genetic loci. Despite this uncertainty, the commercial sector is marketing genotyping of DNA with a view to offering risk profiling to offer targeted screening [96].

Individually, germline risk SNPs may only contribute a limited amount in predicting a person's risk of prostate cancer; however in combination with other factors such as biochemical markers and MRI, these SNPs may add more weight to this prediction. For any individual the total numbers of germline risk SNPs that are present can be combined in a simple multiplicative model that can be used to calculate risk prediction [68]. If this model is applied using the current prostate cancer risk SNPs, men who are in the top 1% risk are estimated to have a 5.7-fold relative risk compared with the average of the general population [71,95].

The incorporation of prostate risk SNPs into a genetic model has been utilized by a paper published by MacInnis *et al*. in 2011 [97]. This model has been designed to use two components to calculate risk; one is 26 prostate cancer risk SNPs (which were available at the time of publication) and a residual polygenic component which incorporates postulated but unknown genetic variants. The results using this model will enable individual men to be stratified into different cumulative risk groups of developing prostate cancer using individual germline SNP risk. The algorithm can be updated and used if new discoveries of SNPs are made [97].

## Screening for prostate cancer

Prostate cancer screening currently consists of measurement of the PSA, with or without clinical examination of the prostate by digital rectal examination. In the USA, the US Preventative Services Task Force did not recommend population-based screening using PSA in 2012 [98]. In the UK the Department of Health have also not recommended population screening, but men can ask to have a PSA measured after counseling of the implications of performing the test by the primary care provider [99].

The recommendations above are based on two large screening studies, which have published conflicting results. The European Randomized Study of Screening for Prostate Cancer (ERSPC) randomized men into two groups; one group was invited for PSA screening and the other group was a control and was not invited for PSA testing. The updated 14-year results of this study showed a 21% reduction in prostate cancer mortality in the screening group [100]. In the updated analysis in the Göteberg cohort, 12 men needed to be treated to prevent one prostate cancer death [101]. In contrast the US Prostate, Lung, Colorectal and Ovarian Cancer Screening Trial (PLCO) failed to demonstrate a benefit in prostate cancer screening using PSA [102]. The main issue with prostate cancer screening is there is an increased frequency of diagnosis of men with indolent prostate cancer that is unlikely to progress to metastatic disease and shorten the life expectancy of the patient. Overtreatment of this group of men can lead to significant lifelong toxicity such as impotence and urinary frequency without the benefit of prolonging survival from cancer.

In order to overcome the issue of overtreatment of aggressive disease it would be useful to have a predictive tool that would be able to differentiate the difference between aggressive and indolent prostate cancer. The correlation between aggressive prostate cancer and germline risk SNPs was investigated in the iCOGs GWAS, where aggressive disease was defined as Gleason ≥ 8 with a PSA ≥100 ng/ml at diagnosis [95]. Some germline risk SNPs demonstrated differing odds ratios in aggressive and nonaggressive disease (although they were in the same direction), which may indicate that there may be certain genetic profiles that would be able to differentiate between aggressive and nonaggressive disease [95]. One way of further validating this would be to perform a case control GWAS, where the control was that of indolent disease and the cases are of aggressive disease to able to find further genetic signatures to help guide a better selection of aggressive cases of prostate cancer that would benefit from treatment.

## Prostate cancer treatment & germline risk SNPs

In prostate cancer there are many treatment options available for men; some of the treatments work well for some men but not for others, but the efficacy of any treatment cannot be predicted before treatment is given. There is also wide variation of treatment toxicity between patients; again this toxicity cannot be predicted before treatment is given. Germline genetic variants have been implicated as perhaps being able to predict treatment responses as well as toxicity, and therefore potentially being able to personalize the treatment to an individual patient.

## Radiotherapy

Radiotherapy is an effective treatment in prostate cancer [103]. Treatment is limited by the dose of radiation that can be tolerated by surrounding normal tissues to the prostate, such as the bowel and the bladder. Treatment toxicity varies significantly between patients, and this cannot be predicted before treatment is delivered. There are some rare variants that cause severe radiation toxicity such as Ataxia Telengectasia, Nijmegen break syndrome, Fanconi's anemia and Bloom's syndrome [104]. These variants are so rare that they cannot be tested on a population level as they do not apply the vast majority of patients.

The initial search for variation in radiation toxicity started with researchers targeting DNA repair pathways in the Fanconi anemia pathway and ataxia telangiectasia pathway, the results have so far been mixed [104,105]. More successful results have been demonstrated by GWAS in radiotherapy cohorts. The first GWAS was performed in African–American set of radiotherapy patients, which discovered a SNP located in the follicle stimulating hormone receptor gene which was associated with an increased incidence of erectile dysfunction [106]. The same group undertook a GWAS in men of European ancestry and found 12 SNPs associated with erectile dysfunction [107]. A further study showed that urinary toxicity was associated with SNPs at 9q21 [108]. A larger GWAS was undertaken in the cohort of patients who entered into The Radiogenomics Study: Assessment of Polymorphisms for Predicting the Effects of Radiotherapy (RAPPER) did not find any SNPs that were associated with increased toxicity after breast and prostate radiotherapy {Barnett, 2012 #205}. The study only contained 637 men which the authors concluded were too small in number to have the power to detect SNPs that may be associated with prostate cancer. In order to address this issue the Radiogenomics Consortium was created to pool together resources and patient numbers from different research groups to enable to power larger studies to detect genetic variation that may predict for radiation toxicity [109].

## Radical prostatectomy

Approximately one-third of patients progress after radical prostatectomy and require adjuvant radiotherapy and some also receive hormonal therapy as well [110]. Tri-modality treatment has significant co-morbidities, and could be spared if these patients who are likely to progress after surgery could be predicted. Candidate gene approaches have been used to try and identify genes that may be related to progression after surgery. Some of the genes that have been discovered include matrix metalloprotineases (*MMP*), *Wnt* signaling pathway genes, androgen receptor genes, *KLK, Bcl2, RNASEL* and Toll like receptors [110–118]. Several GWAS have found germline risk SNPs that have correlated with surgical outcome. Germline risk SNPs have been reported at 8q24 and 10q11 which are associated with biochemical recurrence post radical prostatectomy, and also a further SNP at 8q24 which has been associated with a higher pathological tumor stage and early recurrence post-surgery in African–Americans [8,119]. Other studies in this area have found no relation of surgical outcomes and germline risk SNPs [120]. Therefore further studies and validation are needed to investigate the role of germline risk SNPs and surgical outcomes.

## Androgen deprivation therapy

Androgen deprivation therapy, in the form of luteinizing hormone releasing hormone analogues, antiandrogens and estogens, is an important treatment tool in the treatment of prostate cancer both in the curative and palliative settings [121]. The candidate gene approach has implicated many genes that are involved in the androgen receptor pathway that has led to resistance to hormonal therapy. Some examples of these genes include *SRC* (encoding proto-onocgene tyrosine-protein kinase Src), the androgen receptor gene and *SLCO2B1* and *SLCO1B3* (family of androgen transporters) [117,122–124].

When the androgen receptor is activated it binds to the androgen response elements (ARE) in the nucleus which regulates gene transcription which is an essential part of the prostate cancer pathway. In a recent study 55 SNPs related to ARE were investigated in 601 men receiving androgen deprivation therapy, and some of these SNPs predicted to better survival in this cohort [125]. Disappointingly another study has been shown no correlation between SNPs and the ARE [126].

## Chemotherapy

Docetaxel chemotherapy is commonly used in metastatic prostate cancer after the development of castration resistance [127]. Studies have shown that germline genetic variants have been associated with worse chemotherapy toxicity and poorer clinical outcomes [128,129]. Resistance to chemotherapy has been associated with high expression of the CLUSTERIN gene on chromosome 8q21 [130]. This resistance was reversed when an antisense inhibitor was delivered in combination with docetaxel, which led to improvement in survival [130]. There are no studies involving germline risk SNPs.

## Prostate cancer prevention

The strongest risk factors that show association with development of prostate cancer are age, race and family history, none of which are modifiable. There have been some risk factors that have been identified which potentially could be adjusted. Epidemiological studies have shown that diets rich in dairy and meet products have shown an increased risk of prostate cancer [131,132]. Lycopene which is found in tomatoes has been shown to be protective [133].

There have been two large chemoprevention trials in the USA in Prostate cancer. One of the trials, called the Prostate Cancer Prevention Trial, investigated the use of five-alpha reductase inhibitor finasteride as a chemoprevention agent [134]. It is postulated that finasteride reduces androgen levels. The Prostate Cancer Prevention Trial results showed that there were fewer cancers in those who received finasteride, however the patients that did develop cancer were of a higher grade [134]. This trial was repeated with a similar drug called dutesteride, in which the trial revealed a 22.8% relative reduction in prostate cancer [135]. The US FDA did not approve the use of these drugs in chemoprevention, as the reviewers felt that the antiandrogen action of these drugs merely delayed the detection of tumors [136]. The second large chemoprevention trial used the antioxidants vitamin E and selenium in the SELECT trial [12]. The results of the SELECT trial showed no reduced incidence in prostate cancer. Even though these chemoprevention trials have been negative, there may be a subset of patients with specific genetic profiles who may benefit from intervention which needs to be investigated further.

## Conclusion

Globally prostate cancer is a huge healthcare burden due to its high incidence and prevalence [1]. Early detection of prostate cancer using screening studies have so far been inconclusive, and therefore screening is not recommended in the USA and UK [98,99]. Currently treatment algorithms for prostate cancer do not utilize any germline variants for precision or personalization when deciding about treatment efficacy or toxicity. Over the last decade GWAS and post GWAS studies have shown that there are a number of risk variants that increase the risk of cancer development, and it is estimated that so far only 33% of the familial risk can be explained [71]. Recent published evidence in patients with germline *BRCA* mutations shows that their outcomes are worse than noncarriers when treated with conventional surgery or radiotherapy [137]. With better functional understanding of the biology of the genetic variants as well as large consortia investigating the role of these variants in different patient cohorts it is hoped that more refined models can be developed that can personalize as well as offer better precision in addition to current diagnostic and treatment strategies.

## Future perspective

The ultimate goal of genetic testing would be the potential to provide personalized medicine to an individual patient. This will involve defining risk for every individual so optimal screening can be performed, and also delivering interventions to selected individuals to reduce risk of cancer development. Another further area would be to discover the pathways in which genetic variants can lead to cancer and thus deliver interventions or drugs that interfere with this, with minimum toxicity. In order to discover these genetic variants large samples sizes are needed which can only be achieved in large global consortia (Figure 2). Further benefits of these large consortia are that different clinical cohorts and expertise can be pooled together.In order to answer these questions the NIH funded a large post-GWAS study called Elucidating Loci Involved in Prostate Cancer Susceptibility (ELLIPSE) [138]. This is a grant that focuses on the translational aspects of three projects that were devised to be totally integrated. Project 1 aims are to use the large case control study to discover further SNPs especially in cohorts of Afro-Caribbean origin and those that present with localized or advanced disease. Project 2 aims to investigate the functional aspect of the germline risk SNPs to generate pathways that could potentially be therapeutic targets. Project 3 aims to combine genetic, clinical and epidemiological data from various clinical studies in order to develop risk models, further understand the relationship of genetic variants on screening, chemoprevention and treatment response and toxicity.

**Executive summary**

Evidence from ethnicity data, family history and epidemiological studies suggests a genetic predisposition to prostate cancer.
Genome-wide association studies (GWAS) have identified 100 common germline genetic variants associated with prostate cancer development.
GWAS have been performed to look at the association of germline variants in the outcome in various prostate treatment cohorts.
These germline variants could be used for large population screening of prostate cancer and also enable risk stratification.
The functional elements of these germline variants are still under investigation.
Large international collaborations have been set up to facilitate the answering of complex genetic-epidemiological and genetic-clinical questions.
