# Supplementary material for: Germline genetic profiling in prostate cancer: latest developments and potential clinical applications
Source: Future Sci OA. 2015 Dec 18;2(1):FSO87. doi: 10.4155/fso.15.87 (PMC5137984; doi:10.4155/fso.15.87)
Supplement: Supplementary file 1 [file fso-02-87-s1.docx]

Supplementary Table 1- Showing common susceptibility loci identified in GWAS and recent meta-analysis.

| Locus | SNP | Effect allele | Major Allele | Per allele OR* | Nearby genes | Reference |
| --- | --- | --- | --- | --- | --- | --- |
| 3p12 | rs2660753 | C | T | 1.13 (1.08-1.19) |  | [[47](#_ENREF_47)] |
| 6q25 | rs9364554 | C | T | 1.10 (1.06-1.14) | SLC22A3 | [[47](#_ENREF_47)] |
| 7q21 | rs6465657 | T | C | 1.10 (1.07-1.13) | LMTK2 | [[47](#_ENREF_47)] |
| 10q11 | rs10993994 | C | T | 1.24 (1.20-1.28) | MSMB | [[47](#_ENREF_47)] |
| 11q13 | rs7931342 | G | G | 1.20 (1.16-1.23) |  | [[47](#_ENREF_47)] |
| 19q13 | rs2735839 | G | G | 1.23 (1.18-1.30) | KLK2/3 | [[47](#_ENREF_47)] |
| Xp11 | rs5945619 | T | C | 1.28 (1.21-1.35) | NUDT11 | [[47](#_ENREF_47)] |
| 2p21 | rs1465618 | C | T | 1.07 (1.04-1.11) | THADA | [[47](#_ENREF_47)] |
| 2q31 | rs12621278 | A | A | 1.33 (1.25-1.43) | ITGA6 | [[47](#_ENREF_47)] |
| 4q22 | rs17021918 | C | C | 1.14 (1.10-1.18) | PDLIM5 | [[47](#_ENREF_47)] |
| 4q22 | rs12500426 | C | A | 1.10 (1.06-1.13) | PDLIM5 | [[47](#_ENREF_47)] |
| 4q24 | rs7679673 | C | C | 1.15 (1.11-1.18) | TET2 | [[47](#_ENREF_47)] |
| 8p21 | rs2928679 | G | A | 1.04 (1.01-1.07) | SLC25A37 | [[47](#_ENREF_47)] |
| 8p21 | rs1512268 | C | T | 1.13 (1.10-1.17) | NKX3.1 | [[47](#_ENREF_47)] |
| 11p15 | rs7127900 | G | A | 1.23 (1.18-1.28) |  | [[47](#_ENREF_47)] |
| 22q13 | rs5759167 | G | G | 1.19 (1.15-1.22) |  | [[47](#_ENREF_47)] |
| 8q24 |  rs10086908 | T | T | 1.15 (1.06-1.23) |  | [[72](#_ENREF_72)] |
| 8q24 | rs12543663 | A | C | 1.08 (1.00-1.16) |  | [[72](#_ENREF_72)] |
| 8q24 |  rs620861 | C | C | 1.11 (1.04-1.19) |  | [[72](#_ENREF_72)] |
| 19q13 | rs11672691 | G | A | 1.08 (1.05-1.12) | PCAT19 | [[70](#_ENREF_70), [71](#_ENREF_71)] |
| 2p11 | rs10187424 | T | T | 1.09 (1.06-1.12) |  | [[66](#_ENREF_66)] |
| 2q37 | rs7584330 | A | G | 1.06 (1.02-1.09) | MLPH | [[60](#_ENREF_60), [66](#_ENREF_66)] |
| 3q23 | rs6763931 | G | A | 1.04 (1.01-1.07) | ZBTB38 | [[66](#_ENREF_66)] |
| 3q26 | rs10936632 | A | A | 1.11 (1.08-1.14) |  | [[66](#_ENREF_66)] |
| 5p12 | rs2121875 | A | C | 1.05 (1.02-1.08) | FGF10 | [[66](#_ENREF_66)] |
| 5p15 | rs2242652 | C | C | 1.15 (1.11-1.19) | TERT | [[65](#_ENREF_65), [66](#_ENREF_66)] |
| 5p15 | rs2853676 | C | T | 1.09 (1.05-1.12) | TERT | [[65](#_ENREF_65)] |
| 5p15 | rs2736107 | C | T | 1.12 (1.08-1.15) | TERT | [[65](#_ENREF_65)] |
| 5p15 | rs13190087 | A | C | 1.20 (1.12-1.29) | TERT | [[65](#_ENREF_65)] |
| 6p21 | rs130067 | T | G | 1.05 (1.02-1.09) | CCHCR1 | [[66](#_ENREF_66)] |
| 12q13 | rs10875943 | T | C | 1.07 (1.04-1.10) |  | [[66](#_ENREF_66)] |
| Xq12 | rs5919432 | T | C | 1.06 (1.02-1.12) | AR | [[66](#_ENREF_66)] |
| 1q21 | rs1218582 | A | G | 1.06 (1.03-1.09) |  | [[68](#_ENREF_68)] |
| 1q32 | rs4245739 | A | A | 1.10 (1.05-1.14) |  | [[68](#_ENREF_68)] |
| 2p25 | rs11902236 | C | T | 1.07 (1.03-1.10) |  | [[68](#_ENREF_68)] |
| 2q37 | rs3771570 | C | T | 1.12 (1.08-1.17) | FARP2 | [[68](#_ENREF_68)] |
| 3q13 | rs7611694 | A | A | 1.10 (1.08-1.14) |  | [[68](#_ENREF_68)] |
| 4q13 | rs1894292 | G | G | 1.10 (1.06-1.12) |  | [[68](#_ENREF_68)] |
| 5q35 | rs6869841 | C | T | 1.07 (1.04-1.11) |  | [[68](#_ENREF_68)] |
| 6p21 | rs3096702 | G | A | 1.07 (1.04-1.10) | NOTCH4 | [[68](#_ENREF_68)] |
| 6q21 | rs2273669 | A | G | 1.07 (1.03-1.11) |  | [[68](#_ENREF_68)] |
| 6q25 | rs1933488 | A | A | 1.12 (1.09-1.15) |  | [[68](#_ENREF_68)] |
| 7p15 | rs12155172 | G | A | 1.11 (1.07-1.15) | SP8 | [[68](#_ENREF_68)] |
| 8p21 | rs11135910 | C | T | 1.11 (1.07-1.16) | EBF2 | [[68](#_ENREF_68)] |
| 10q24 | rs3850699 | A | A | 1.10 (1.06-1.12) | TRIM8 | [[68](#_ENREF_68)] |
| 11q22 | rs11568818 | T | T | 1.10 (1.06-1.14) | MMP7 | [[68](#_ENREF_68)] |
| 12q24 | rs1270884 | G | A | 1.07 (1.04-1.10) | TBX5 | [[68](#_ENREF_68)] |
| 14q22 | rs8008270 | C | C | 1.12 (1.08-1.16) | FERMT2 | [[68](#_ENREF_68)] |
| 14q24 | rs7141529 | T | C | 1.09 (1.06-1.12) | RAD51B | [[68](#_ENREF_68)] |
| 17p13 | rs684232 | T | C | 1.10 (1.07-1.14) |  | [[68](#_ENREF_68)] |
| 17q21 | rs11650494 | G | A | 1.15 (1.09-1.22) |  | [[68](#_ENREF_68)] |
| 18q23 | rs7241993 | C | C | 1.09 (1.05-1.12) |  | [[68](#_ENREF_68)] |
| 20q13 | rs2427345 | C | C | 1.06 (1.03-1.10) |  | [[68](#_ENREF_68)] |
| 20q13 | rs6062509 | T | T | 1.12 (1.09-1.16) | ZGPAT | [[68](#_ENREF_68)] |
| Xp22 | rs2405942 | A | A | 1.14 (1.09-1.20) |  | [[68](#_ENREF_68)] |
| 1q21 | rs17599629 | A | G | 1.10 (1.07-1.13) | GOLPH3L | [[69](#_ENREF_69)] |
| 2p25 | rs9287719 | T | C | 1.07 (1.04-1.09) | NOL10 | [[69](#_ENREF_69)] |
| 4q13 | rs10009409 | C | T | 1.09 (1.06-1.12) |  | [[69](#_ENREF_69)] |
| 6p21 | rs3129859 | G | G | 1.08 (1.06-1.11) | HLA-DRA | [[69](#_ENREF_69)] |
| 6p22 | rs7767188 | G | A | 1.08 (1.06-1.11) | TRIM31 | [[69](#_ENREF_69)] |
| 6p24 | rs4713266 | C | C | 1.08 (1.04-1.09) | NEDD9 | [[69](#_ENREF_69)] |
| 7p12 | rs56232506 | G | A | 1.07 (1.05-1.09) | TNS3 | [[69](#_ENREF_69)] |
| 9p21 | rs17694493 | C | G | 1.10 (1.06-1.13) | CDKN2B-AS1 | [[69](#_ENREF_69)] |
| 10q11 | rs76934034 | T | T | 1.14 (1.10-1.18) | Mar-08 | [[69](#_ENREF_69)] |
| 11q23 | rs11214775 | G | G | 1.08 (1.05-1.11) | HTR3B | [[69](#_ENREF_69)] |
| 12q13 | rs80130819 | A | A | 1.12 (1.08-1.18) |  | [[69](#_ENREF_69)] |
| 14q24 | rs8014671 | G | G | 1.08 (1.05-1.10) |  | [[69](#_ENREF_69)] |
| Xp11 | rs2807031 | T | C | 1.07 (1.04-1.09) | XAGE3 | [[69](#_ENREF_69)] |
| Xq13 | rs6625711 | T | A | 1.07 (1.05-1.08) |  | [[69](#_ENREF_69)] |
| Xq13 | rs4844289 | A | G | 1.05 (1.04-1.07) |  | [[69](#_ENREF_69)] |
| 1q32 | rs1775148 | T | C | 1.06 (1.03-1.08) | SLC41A1 | [[69](#_ENREF_69)] |
| 6q14 | rs9443189 | A | G | 1.07 (1.04-1.11) | MYO6 | [[69](#_ENREF_69)] |
| 14q23 | rs7153648 | G | C | 1.09 (1.04-1.13) | SIX1 | [[69](#_ENREF_69)] |
| 16q22 | rs12051443 | G | A | 1.06 (1.03-1.08) | PHLPP2 | [[69](#_ENREF_69)] |
| 20q13 | rs12480328 | T | T | 1.14 (1.08-1.18) | ADNP | [[69](#_ENREF_69)] |
| 21q22 | rs1041449 | A | G | 1.06 (1.04-1.09) | TMPRSS2 | [[69](#_ENREF_69)] |
| 22q11 | rs2238776 | G | G | 1.09 (1.06-1.12) | TBX1 | [[69](#_ENREF_69)] |
| 1p35 | rs636291 | G | A | 1.18(1.12-1.24) | PEX14 | [[69](#_ENREF_69)] |
| 2p15 | rs721048 | G | A | 1.12 (1.07-1.16) |  | [[67](#_ENREF_67)] |
| 3q21 | rs10934853 | C | A | 1.12 (1.08-1.16) | EEFSEC | [[54](#_ENREF_54)] |
| 7p15 | rs10486567 | G | G | 1.18 (1.12-1.22) | JAZF1 | [[58](#_ENREF_58)] |
| 8q24 | **rs1447295** | C | **A** | 1.42 (1.35-1.49) |  | **[**[**64**](#_ENREF_64)**]** |
| 8q24 | **rs6983267** | G | **G** | 1.22 (1.19-1.27) |  | **[**[**57**](#_ENREF_57)**]** |
| 8q24 | **rs16901979** | C | **A** | 1.55 (1.43-1.68) |  | **[**[**62**](#_ENREF_62)**]** |
| 9q33 | **rs1571801** | G | **T** | 1.27 (1.10-1.48) | **DAB2IP** | **[**[**63**](#_ENREF_63)**]** |
| 10q26 | **rs4962416** | T | **C** | 1.04 (1.00-1.09) | **CTBP2** | **[**[**58**](#_ENREF_58)**]** |
| 12q13 | **rs902774** | G | **A** | 1.17 (1.11-1.24) | **KRT8** | **[**[**60**](#_ENREF_60)**]** |
| 17q12 | **rs4430796** | A | **A** | 1.22 (1.19-1.27) | **HNF1B** | **[**[**61**](#_ENREF_61)**]** |
| 17q12 | **rs11649743** | G | **G** | 1.14 (1.10-1.19) | **HNF1B** | **[**[**59**](#_ENREF_59)**]** |
| 17q24 | **rs1859962** | T | **G** | 1.19 (1.14-1.23) |  | **[**[**55**](#_ENREF_55)**,** [**61**](#_ENREF_61)**]** |
| 19q13 | **rs8102476** | C | **C** | 1.12 (1.08-1.15) |  | **[**[**54**](#_ENREF_54)**]** |
| 22q13 | **rs9623117** | T | **C** | 1.11 (1.04-1.19) | **TNRC6B** | **[**[**52**](#_ENREF_52)**]** |
| 2p24 | **rs13385191** | G | **G** | 1.15 (1.10-1.21) | **C2orf43** | **[**[**51**](#_ENREF_51)**]** |
| 3p11 | rs2055109 | T | C | 1.20 (1.13-1.29) |  | [[56](#_ENREF_56)] |
| 5p15 | rs12653946 | C | T | 1.26 (1.20-1.33) | IRX4 | [[51](#_ENREF_51)] |
| 6p21 | rs1983891 | C | T | 1.15 (1.09-1.21) | FOXP4 | [[51](#_ENREF_51)] |
| 6q22 | rs339331 | T | T | 1.22 (1.15-1.28) | RFX6 | [[51](#_ENREF_51)] |
| 9q31 | rs817826 | T | C | 1.41 (1.29-1.54) | RAD23B | [[50](#_ENREF_50)] |
| 10q26 | rs2252004 | C | C | 1.16 (1.10-1.22) |  | [[56](#_ENREF_56)] |
| 17q21 | rs7210100 | G | A | 1.51 (1.35-1.69) | ZNF652 | [[53](#_ENREF_53)] |

*Data for Effect allele frequency and per allele OR (odds ratio) are taken from the original publications. 95% confidence intervals are given in brackets where available.
